# Comparison of minimally invasive transforaminal lumbar interbody fusion and endoscopic lumbar interbody fusion for lumbar degenerative diseases: a retrospective observational study

**DOI:** 10.1186/s13018-023-03875-6

**Published:** 2023-05-27

**Authors:** Hao Chen, Goudi Zheng, Zhenyu Bian, Changju Hou, Maoqiang Li, Zhen Zhang, Liulong Zhu, Xuepeng Wang

**Affiliations:** Department of Orthopedics Surgery, Affiliated Hangzhou First People’s Hospital, Zhejiang University School of Medicine, Hangzhou, 310006 China

**Keywords:** Minimally invasive transforaminal lumbar interbody fusion, Endoscopic lumbar interbody fusion, Lumbar degenerative diseases

## Abstract

**Background:**

Minimally invasive transforaminal lumbar interbody fusion (MIS-TLIF) and endoscopic lumbar interbody fusion (Endo-LIF) are both minimally invasive interbody fusion procedures for lumbar degenerative diseases. In this study, we attempted to compare the clinical efficacy and postoperative outcomes of MIS-TLIF and Endo-LIF for lumbar degenerative diseases.

**Methods:**

The study cohort comprised 99 patients with lumbar degenerative diseases treated by MIS-TLIF or Endo-LIF from January 2019 to July 2021. The clinical outcomes (visual analogue scale (VAS), Oswestry disability index (ODI), and MacNab criteria) preoperatively, 1 month postoperatively, 3 months postoperatively, and 1 year postoperatively were compared between the two groups.

**Results:**

There were no significant differences between the two groups in sex, age, disease duration, affected spine segment, and complications (*P* > 0.05). The operation time was significantly longer in the Endo-LIF group than the MIS-TLIF group (155.25 ± 12.57 vs. 123.14 ± 14.50 min; *P* < 0.05). However, the Endo-LIF group had a significantly smaller blood loss volume (61.79 ± 10.09 vs. 259.97 ± 14.63 ml) and shorter hospital stay (5.46 ± 1.11 vs. 7.06 ± 1.42 days) than the MIS-TLIF group. In both groups, the ODI and VAS scores for lower back pain and leg pain were significantly lower at each postoperative timepoint than preoperatively (*P* < 0.05). Although there were no significant differences between the two groups in the ODI and VAS scores for lower back pain and leg pain (*P* > 0.05), the VAS for lower back pain was lower in the Endo-LIF group than the MIS-TLIF group at each postoperative timepoint. The MacNab criteria showed that the improvement rate was 92.2% in the MIS-TLIF group and 91.7% in the Endo-LIF group, with no significant difference between the two groups (*P* > 0.05).

**Conclusions:**

There were no significant differences in short-term surgical outcomes between the MIS-TLIF and Endo-LIF groups. Compared with the MIS-TLIF group, the Endo-LIF group incurred less damage to surrounding tissues, experienced less intraoperative blood loss, and had less lower back pain, which is more conducive to recovery.

## Background

Lumbar interbody fusion is effective for the treatment of lumbar degenerative diseases, such as lumbar spinal stenosis, lumbar disc herniation, and lumbar spondylolisthesis. Typically, open interbody fusion is considered the preferred therapy. With the development of posterior lumbar interbody fusion and transforaminal lumbar interbody fusion (TLIF) in the 1950s, the posterior approach has been widely recognized as a typical surgical procedure [1–3]. However, the posterior approach is still unacceptable to many patients due to the significant pain, tissue damage, and postoperative complications associated with open surgery [4].

Advances in surgical techniques have led to the introduction of minimally invasive TLIF (MIS-TLIF) [5]. MIS-TLIF is considered an effective alternative to open surgery, as the paravertebral structures are adequately protected under the channel. Compared with traditional open surgery, MIS-TLIF has the advantages of less trauma, less bleeding, and faster recovery [6–8]. However, MIS-TLIF also has the disadvantages of a limited working space and field of vision, and the problem of tissue damage due to the use of dilating tubes that may cause extrusion of the surrounding muscles [9].

In recent years, with the widespread use of endoscopic techniques, endoscopic lumbar interbody fusion (Endo-LIF) has become a new treatment option for lumbar degenerative diseases [10]. The efficacy of Endo-LIF in minimizing tissue damage, reducing blood loss, and lessening pain has been well reported [11–13]. Endo-LIF is the basis for other endoscopic fusion procedures. However, in contrast to other endoscopic fusion procedures through posterolateral approach like Endoscopic-TLIF (endoscopic transforaminal lumbar interbody fusion, Endo-TLIF), Endo-LIF does not require the removal of the superior and inferior facet joints, or removes a little amount of superior articular process, and enables the surgeon to reach the disc directly through Kambin’s triangle to decompress the area [14]. Therefore, this new technique theoretically causes less muscle damage and less intraoperative blood loss and facilitates the preservation of posterior structures compared with MIS-TLIF. However, there is no objective evidence comparing the clinical outcomes of the two fusion techniques. The aim of the present study was to compare MIS-TLIF and Endo-LIF to demonstrate the clinical efficacy of Endo-LIF for degenerative diseases.

## Methods

### Patient characteristics

We retrospectively reviewed 99 patients surgically treated for lumbar degenerative disease from January 2019 to July 2021. Among these patients, 51 received MIS-TLIF and 48 received Endo-LIF. The surgeries were performed by two fellowship-trained surgeon groups with extensive experience separately. The cohort comprised 56 men and 43 women with a mean age of 57.6 years (range 45–73 years) and a mean disease duration of 16.2 months (range 7–23 months). The lumbar degenerative levels were L3/4 in 14 patients, L4/5 in 21 patients, L5/S1 in 26 patients, L3–L5 in 17 patients, L4–S1 in 18 patients, and L2–L5 in 3 patients. There were no significant differences between the MIS-TLIF and Endo-LIF groups in sex, age, duration of disease, and degenerative levels (Table 1; *P* > 0.05).

**Table 1:** Patient basic information

	MIS-TLIF group	Endo-LIF group	*P*
Gender (male:female**)**	30:21	26:22	0.322
Age (y)	57.32 ± 7.10	57.65 ± 7.89	0.908
Levels			0.974
L3/4	7	7	> 0.05
L4/5	11	10	
L5/S1	14	12	
L3–L5	8	9	
L4–S1	9	9	
L2–L5	2	1	
Disease duration	15.88 ± 4.14	16.10 ± 4.09	0.399
Clinical diagnosis			> 0.05
Lumbar disc herniation	14	11	
Lumbar spondylolisthesis	12	11	
Lumbar spinal stenosis	25	26	

### Inclusion and exclusion criteria

The inclusion criteria were (1) persistent neurological symptoms after more than 3 months of conservative therapy; (2) lumbar disc herniation with instability; (3) degree I or II spondylolisthesis based on radiography, CT, and MRI; (4) foraminal stenosis or central canal stenosis; (5) cartilage endplate inflammation.

The exclusion criteria were (1) previous lumbar surgical treatment; (2) severe spinal deformity; (3) severe lumbar spinal stenosis or severe lumbar instability with spondylolisthesis greater than degree II; (4) severe underlying disease that prevented surgical treatment; (5) tumour, infection, or severe osteoporosis; (6) loss of vertebral space height greater than 30%; (7) unwillingness or inability to participate in treatment and complete follow-up.

### Surgical techniques

#### MIS-TLIF group

**Fig. 1:**
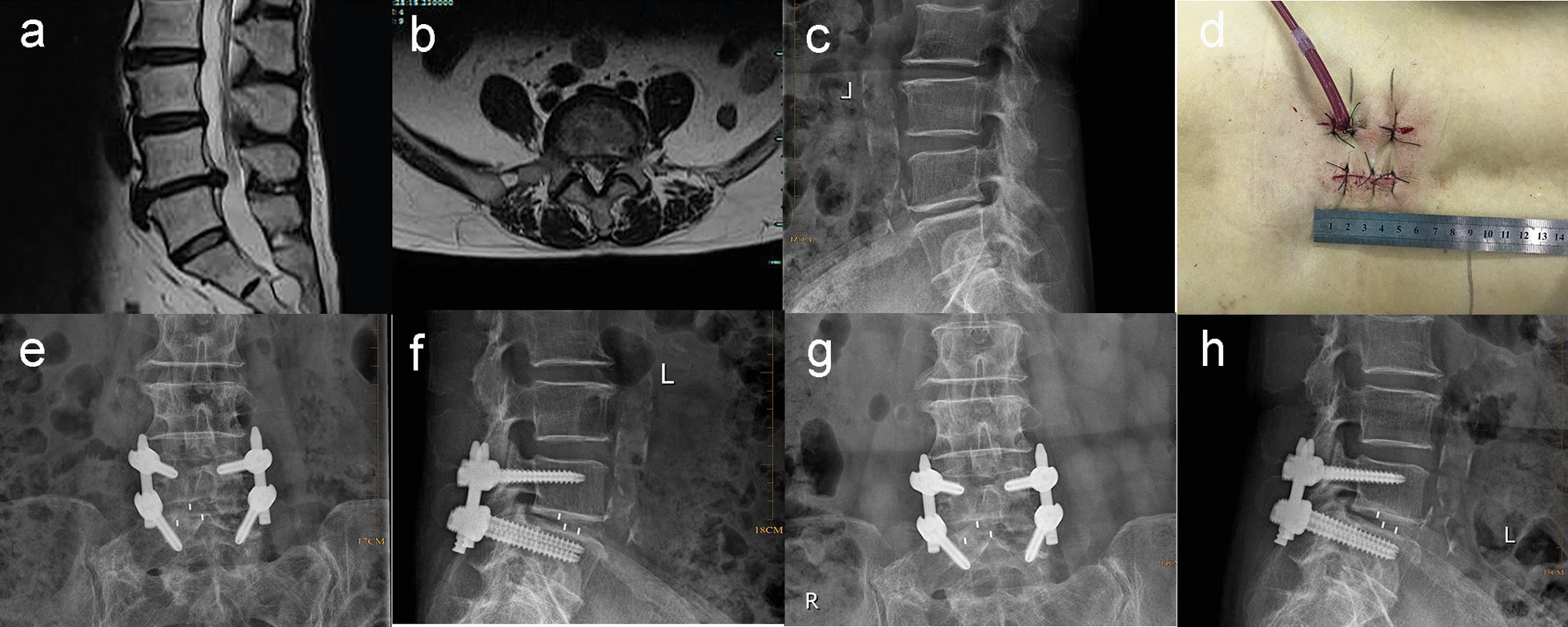
Typical case 1. A 66-year-old female with low back pain and left lower limb radiating pain for 2 years, aggravating for 6 months. Sagittal and axial MRI show disc herniation at L4/5 level (**a**, **b**). Lateral X-ray demonstrates reduced intervertebral height at L4/5 level (**c**). Postoperative surgical incision (**d**). Anteroposterior and lateral X-ray one month after surgery (**e**, **f**). Anteroposterior and lateral X-ray 1 year after surgery (**g**, **h**)

The patient was placed in the prone position under general anaesthesia. The target disc space and location of the incision were identified using C-arm fluoroscopy. A skin incision of 3–4 cm was created just lateral to the midline. After fully exposing the superior and inferior facet joints of the diseased segment, the inferior facet and medial margins of the superior facet were removed with rongeur forceps. If necessary, part of the superior margin of the inferior lamina was removed to completely decompress the spinal canal and nerve root canal. Discectomy and endplate preparation was performed with a shaver. The inferior and superior facets were removed as bone particles and mixed with allogeneic bone before a cage was inserted into the intervertebral space (Fig. 1). Finally, the pedicle screws were inserted percutaneously and longitudinal titanium rods were attached.

#### Endo-LIF group

**Fig. 2:**
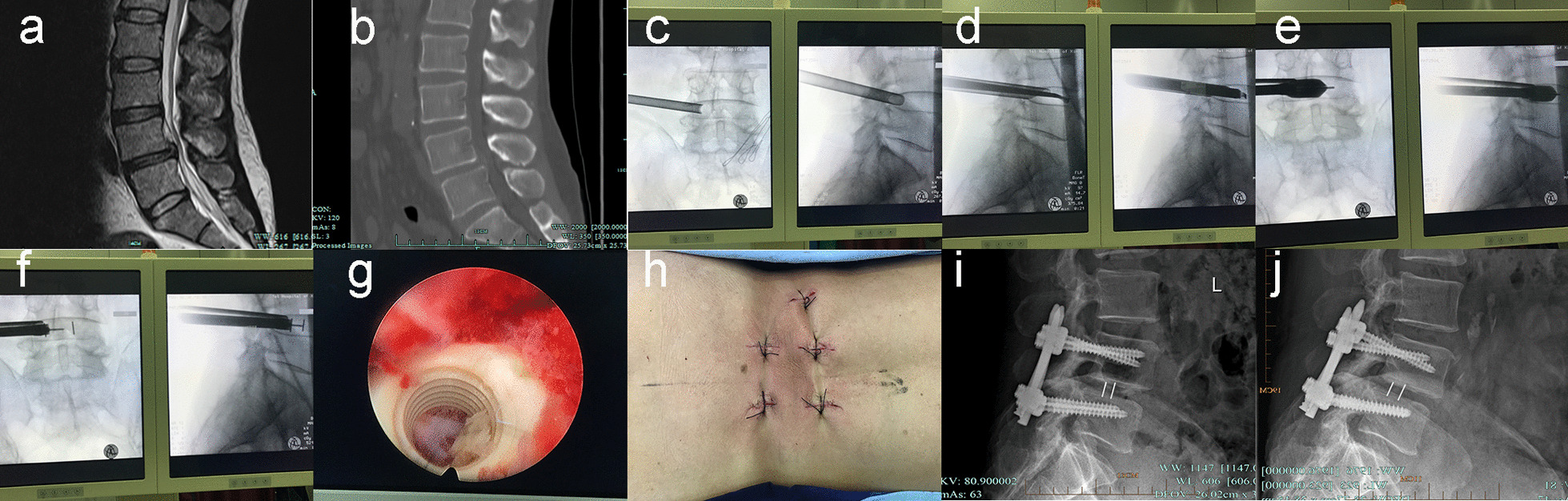
Typical case 2. A 53-year-old male with recurrent low back pain accompanied by right lower limb radiating numbness and pain for six months, aggravating for 10 days. Sagittal MRI and CT show disc herniation and instability at L4/5 level (**a**, **b**). Achieve the foraminoplasty and laminectomy to have enough room for decompression (**c**). Clear the disc space with a scraper (**d**). Test and adjust the mould of cage by intraoperative fluoroscopy (**e**). Implant cage with working channel or nerve root retractor (**f**). Perform secondary endoscopic exploration and clear the disc space (**g**). Postoperative surgical incision (**h**). Lateral X-ray one month after surgery (**i**). Lateral X-ray 1 year after surgery (**j**)

**Fig. 3:**
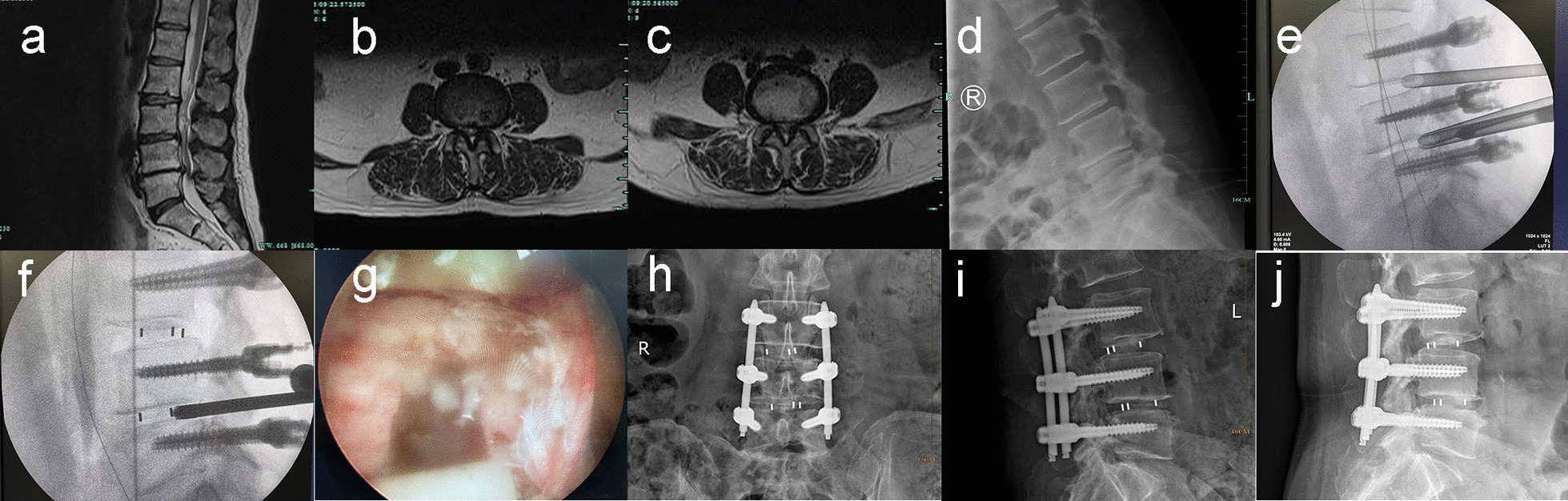
Typical case 3. A 66-year-old female with low back pain for 10 years, aggravating with lower limbs numbness and soreness for six months. Sagittal MRI shows disc herniation at L3/4 and L4/5 levels, and endplate degeneration with reduced intervertebral height at L4/5 level (**a**). Axial MRI show spinal stenosis at L3/4 and L4/5 levels (**b**, **c**). Hyperextension X-ray demonstrates instability at L3/4 and L4/5 levels (**d**). Place the working channel and perform the foraminoplasty (**e**). Implant the cage and then make secondary decompression (**f**, **g**). Anteroposterior and lateral X-ray one month after surgery (**h**, **i**). Lateral X-ray 1 year after surgery (**j**)

**Fig. 4:**
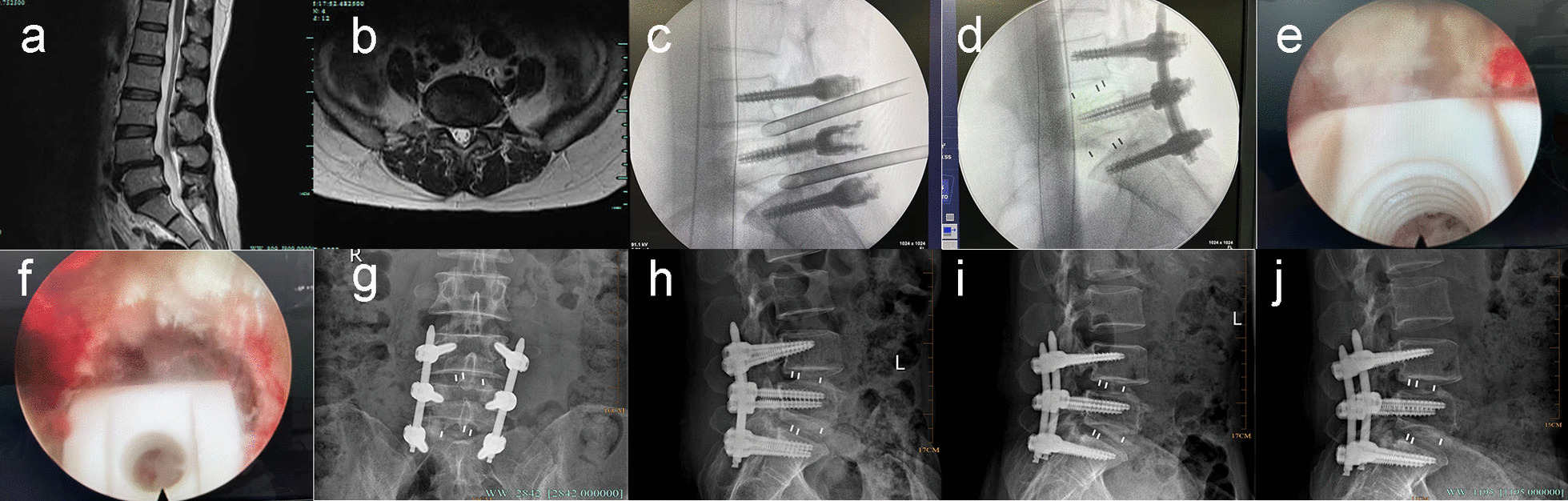
Typical case 4. A 51-year-old female with recurrent low back pain for more than 10 years, aggravating with right lower limb pain and numbness for 3 years. Sagittal MRI shows disc herniation at L5/S1 level and spondylolisthesis at L4/5 level (**a**). Axial MRI shows disc herniation at L5/S1 level (**b**). Place the working channel and perform the foraminoplasty (**c**). Implant the cage (**d**). Make secondary decompression at L4/5 level (**e**) and L5/S1 level (**f**). Anteroposterior and lateral X-ray one month after surgery (**g**, **h**). Lateral X-ray three months after surgery (**i**). Lateral X-ray 1 year after surgery (**j**)

The patient was placed in the prone position under general anaesthesia and the entry point was determined using the Yeung endoscopic spine system technique. The cephalic tilt of the puncture was maintained at an angle of 0°–10°. In the L5/S1 segment, if the puncture site was difficult to access due to iliac crest occlusion or L5 transverse process hypertrophy, the surgeon instead used the Tom needle or reduced the paracentral distance between the entry point and the midline of the spinous process. Care was taken to accurately measure the paracentral distance in the coronal position and the maximum safe angle in the sagittal position preoperatively on MRI and CT of the affected segment. To ensure optimal implantation of the cage, repeated X-ray fluoroscopy was performed to ensure that after the puncture needle had passed through Kambin’s triangle, the end of the needle was located in the anterior 2/3 of the disc in the lateral view and past the spinous process midline in the frontal view. The dilator was placed along the puncture needle and guidewire to establish a working channel between the skin and the affected disc. The ideal situation was a 10–12-mm channel in half of the disc. The foraminotomy was performed using a trephine to remove part of the ventral superior facet and preserve it for bone grafting. The disc tissue and endplate were removed with rongeurs and a scraper (Fig. 2). A nerve retractor was placed dorsally in the channel and offset towards the head of the patient to protect the exiting nerve root and allow more room for manipulation. When possible, the surgeon placed a small amount of allogeneic bone combined with decalcified dental matrix (BMP-2) to reduce the risk of non-fusion. The surgeon then placed the appropriately sized cage under fluoroscopic surveillance and protection with a nerve retractor. Secondary endoscopic exploration of the spinal canal and nerve root cleaning were then performed to ensure that the dura and nerve roots were not compressed (Figs. 3, 4). Finally, the bilateral pedicle screws were inserted percutaneously under fluoroscopy at the affected segment.

### Outcomes

The patients were followed up at 1, 3, and 12 months postoperatively by clinical visits. The surgery duration, intraoperative blood loss volume, duration of hospitalization, postoperative complications, Oswestry Disability Index (ODI), and visual analogue scale (VAS) scores for lower back pain and leg pain were compared between the two groups. A higher VAS score (maximum 10) indicates more severe pain, while a higher ODI score (maximum 50) indicates poorer quality of life. The clinical outcome was evaluated by the MacNab criteria, and fusion rates were assessed on imaging performed at the final follow-up.

### Statistical analysis

All data were statistically analysed using SPSS 19.0 software, and the measures were expressed as mean ± standard deviation. The *t* test was used to compare the means of two independent samples between groups, while ANOVA was used to compare the means of multiple samples. Comparisons of count data between groups were made using the *χ*^2^ test. *P* < 0.05 was considered statistically significant.

## Results

The surgery was completed in all patients. The operative time, intraoperative blood loss volume, hospital stay, postoperative fusion rate, and postoperative complications in both groups are detailed in Tables 2 and 3. The operative time and fluoroscopy time were significantly longer in the Endo-LIF group than the MIS-TLIF group (*P* < 0.05). However, the Endo-LIF group had a significantly smaller intraoperative blood loss volume and significantly shorter hospital stay than the MIS-TLIF group (*P* < 0.05). There was no significant difference in the surgical complications between the two groups (*P* > 0.05).

**Table 2:** Perioperative parameters

	MIS-TLIF group	Endo-LIF group	*P*
Operative time (minute)	123.13 ± 14.50	155.25 ± 12.97	< 0.001
Blood loss (ml)	259.97 ± 14.63	61.79 ± 10.09	< 0.001
Intraoperative fluoroscopy time (second)	29.13 ± 3.05	42.94 ± 21.63	< 0.001
Hospital stays (day)	7.06 ± 1.42	3.46 ± 1.11	< 0.001
Interbody fusion rate	50 (98.0%)	47 (97.9%)	> 0.05

**Table 3:** Complications postoperatively

	MIS-TLIF group	Endo-LIF group	*P*
Residual nucleus pulposus	0	1	0.766
Exiting nerve root injury	0	2	
Traversing nerve root injury	3	0	
Dural tears	3	2	
Infection	0	0	
Non-fusion of the cage	1	1	

**Fig. 5:**
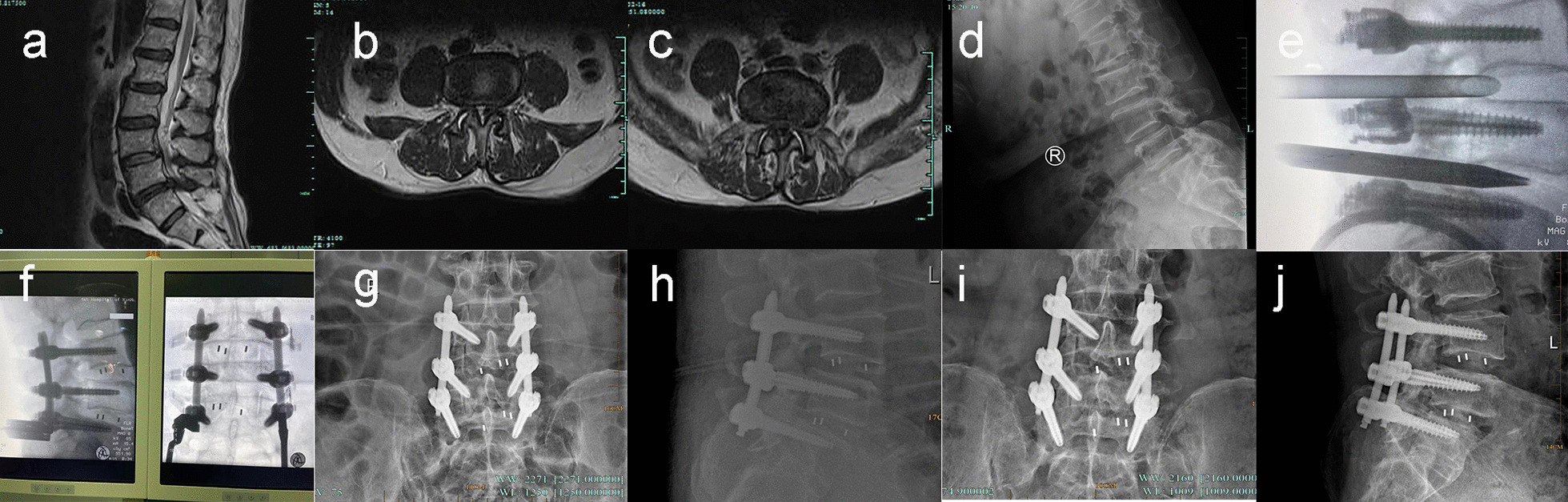
Typical case 5. An 82-year-old male with left hip pain and left lower limb numbness for over a year. Sagittal MRI shows disc herniation at L5/S1 level and spondylolisthesis at L4/5 level (**a**). Axial MRI show severe spinal stenosis at L4/5 and L5/S1 levels (**b**, **c**). Hyperextension X-ray demonstrates instability at L4/5 and L5/S1 levels (**d**). Finish the decompression and implant cage to intervertebral space (**e**, **f**). Anteroposterior and lateral X-ray one month after surgery (**g**, **h**). Anteroposterior and lateral X-ray 1 year after surgery (**i**, **j**)

Patients in both groups were assessed postoperatively for a mean follow-up period of 16.3 months (range 12–25 months). Compared with the preoperative values, there were significant reductions in the VAS scores for lower back pain and leg pain and the ODI at all postoperative timepoints in both groups (*P* < 0.05). Although there were no significant differences between the two groups in the preoperative VAS scores for lower back pain and leg pain, ODI, and postoperative fusion rate (*P* > 0.05), the Endo-LIF group had lower VAS scores for lower back pain than the MIS-TLIF group at all follow-up timepoints. The MacNab criteria at 1 year postoperatively showed that the prevalence of excellent spinal stability was 92.2% in the MIS-TLIF group, including 38 patients with excellent stability, 9 with good stability, and 4 with fair stability; the prevalence of excellent spinal stability in the Endo-LIF group was 91.7%, including 35 patients with excellent stability, 9 with good stability, and 4 with fair stability (Fig. 5). The spinal stability based on the MacNab criteria did not significantly differ between the two groups (Table 4; *P* > 0.05).

**Table 4:** Comparison of follow-up outcomes in group Endo-LIF and group MIS-TLIF

	MIS-TLIF group	Endo-LIF group	*P*
VAS of low-back pain			
Preoperative	5.89 ± 0.68	5.70 ± 0.91	0.057
1 month postoperative	2.48 ± 0.28	2.42 ± 0.33	0.306
3 months postoperative	1.97 ± 0.20	1.90 ± 0.22	0.090
12 months postoperative	1.29 ± 0.17	1.23 ± 0.19	0.167
VAS of leg pain			
Preoperative	5.56 ± 0.53	5.47 ± 0.34	0.347
1 month postoperative	1.28 ± 0.28	1.23 ± 0.21	0.297
3 months postoperative	0.92 ± 0.17	0.86 ± 0.16	0.064
12 months postoperative	0.78 ± 0.12	0.78 ± 0.16	0.972
ODI			
Preoperative	54.88 ± 3.43	54.07 ± 3.28	0.261
1 month postoperative	44.95 ± 3.32	43.66 ± 2.46	0.074
3 months postoperative	20.61 ± 2.33	21.21 ± 2.30	0.167
12 months postoperative	10.87 ± 2.02	11.48 ± 2.15	0.156
MacNab			
12 months postoperative	38:9:4:0	35:9:4:0	> 0.05

*VAS*—visual analogue scale, *ODI*—Oswestry Disability Index

## Discussion

Lumbar degenerative disease is a common disorder that causes various neurological symptoms. With the advances in minimally invasive spinal surgery techniques, minimally invasive surgery has become the preferred treatment for lumbar degenerative disease. Since MIS-TLIF was first proposed by Foley in 2002 [10], it has gained in popularity owing to its great advantages. A comparative study showed that the MIS-TLIF group experienced less injury and recovered sooner after surgery for lumbar degenerative disease than open TLIF [15]. In addition, MIS-TLIF retains the advantage of direct and adequate decompression of the canal, which allows for more adequate nerve decompression through the intervertebral foramen and has a wider range of indications for more complex degenerative lumbar diseases [16]. As a result, MIS-TLIF has become an effective alternative to open surgery in recent years [17, 18].

With the widespread use of endoscopy in spinal therapy, Osman et al. [19] first reported a technique of endoscopic transforaminal decompression, interbody fusion, and percutaneous pedicle screw implantation for lumbar degenerative disc disease, which is known as Endo-LIF. In the study by Osman et al. [19], 29.6% of patients achieved strong fusion and 36.2% had a stable internal fixation system; the reason for the relatively low fusion rate may be related to the absence of an implanted cage and autologous bone. Wang et al. [20] reported 10 cases of endoscopic transforaminal approach interbody fusion, with no intraoperative or postoperative complications and a 100% fusion rate. The authors concluded that Endo-LIF may be an alternative to conventional fusion therapy [20]. Endo-LIF uses a channel through Kambin’s triangle and thus does not require the osseous channel from the posterior or posterolateral approach that is needed in currently emerging endoscopic techniques, like unilateral biportal endoscopy. The absence of the need to remove the facet joint in Endo-LIF reduces the difficulty of the procedure and the probability of iatrogenic nerve injury and instability. Similarly to MIS-TLIF, Endo-LIF has the advantage of being minimally invasive, in addition to other advantages such as a quicker recovery, shorter duration of hospitalization, and lower costs [21]; however, no study has compared the specific clinical outcomes of MIS-TLIF and Endo-LIF.

In our study, we retrospectively compared a group of patients treated with Endo-LIF versus a group treated with MIS-TLIF. Compared with the MIS-TLIF group, the Endo-LIF group had significantly less intraoperative blood loss and a shorter hospital stay, but a longer operative time and fluoroscopy time. Although the VAS scores, ODI, MacNab criteria, and postoperative fusion rate at 1 year postoperatively were similar in both groups, the Endo-LIF group had less trauma to the surrounding tissues and had lower VAS scores for lower back pain than the MIS-TLIF group at all postoperative timepoints. Jung et al. [22] performed a meta-analysis to compare the results of full-endoscopic lumbar interbody fusion and MIS-TLIF for lumbar degenerative disease in a total cohort of 423 patients. The authors concluded that the immediate results of full-endoscopic lumbar interbody fusion were favourable in terms of blood loss and VAS for back pain compared with MIS-TLIF, although there were no differences between the two techniques in complications, short- or medium-term clinical outcomes, and fusion rates. Our findings are in agreeance with the findings of Jung et al. [21]

The complication rate for totally endoscopic lumbar interbody fusion has been reported to be 13.2% (range 0–38.6%) [23]. In our study, two patients had signs of exit nerve root injury after Endo-LIF, and their symptoms were relieved after 1 month of conservative therapy and functional exercise. To reduce this complication, we used a trephine instead of a drill to perform root-forming arthroplasty. In our clinical experience, none of the patients who have undergone this modified procedure have had extrusion of the exit nerve root. In addition, one patient in the Endo-LIF group had residual nucleus pulposus, which may have been due to incomplete microscopic nerve root and endplate treatment; secondary decompression is recommended for this issue. Further endoscopic exploration of the spinal canal and nerve root cleaning should be performed after the cage is inserted to prevent bone and nucleus pulposus from entering the spinal canal. We also noted that one patient in each group had non-fusion of the cage at the 1-year follow-up. We attributed this complication to the following three possible causes. (1) Cage subsidence owing to collapse of the endplate. In the process of propping up the vertebral space and bone grafting, endplate injury is a common complication, especially in osteoporotic patients. Therefore, it is important to match the cage perfectly with the channel. It is advisable to place the corresponding type of cage under fluoroscopic surveillance and protection with a nerve retractor. In addition, attention should be paid to the angle of bone graft entry to prevent accidental injury to the endplate. (2) Incomplete removal of the endplate, which is very likely to lead to non-fusion. In our experience, it is best to use a hook and curette instead of a reamer, as this will clean the endplate efficiently and thoroughly and make full use of the advantages of Endo-LIF to ensure satisfactory endplate cleaning by direct visualization. (3) Inappropriate autologous bone grafting. The choice of bone graft largely influences the postoperative fusion. While a large amount of autologous bone is obtained during MIS-TLIF, only a little of the facet joint is cut during Endo-LIF. Therefore, we empirically used allogeneic bone combined with decalcified dental matrix (BMP-2) in Endo-LIF to effectively shorten the fusion time and improve the success rate of fusion. The other common complications were cerebrospinal fluid leakage and dural tears, which did not occur at a high rate in the present study.

There are still some limitations of Endo-LIF. Firstly, for some severe lumbar degenerative diseases, such as severe spinal stenosis, severe lumbar spondylolisthesis, and foraminal stenosis, MIS-TLIF may be more appropriate because Endo-LIF may not be able to achieve adequate microscopic decompression. Secondly, the operative time was significantly longer in the Endo-LIF group than the MIS-TLIF group. Although endoscopic techniques have improved considerably, the surgical instruments used in Endo-LIF may require a longer operative time. Furthermore, Endo-LIF may require more fluoroscopic examinations to locate the incision and check the placement of the cage, which exposes the Endo-LIF group to a higher dose of radiation than the MIS-TLIF group. Thirdly, the amount of removal of facet joint was less in the Endo-LIF group than the MIS-TLIF group. BMP-2 was added to allogeneic bone grafts for better fusion in the Endo-LIF group, which might lead to statistical bias. Fourthly, Endo-LIF is technically challenging and requires a long learning period. The learning time of the technique and the surgeon’s skills also affect the postoperative recovery and complications [24]. Finally, the number of patients in the two groups was relatively small, and all patients in the study were only followed for less than 25 months. So that, a larger sample with a longer follow-up time are needed to make definitive clinical conclusions.

## Conclusions

The safety and clinical efficacy of MIS-TLIF and Endo-LIF do not differ significantly in the short- or medium-term. The Endo-LIF group has less damage to surrounding tissues, less intraoperative blood loss, and less postoperative back pain, which is better for the patient's recovery in the long-term.

## Data Availability

All data generated or analysed during this study are included in this article.

## Abbreviations

MIS-TLIF: Minimally invasive transforaminal lumbar interbody fusion
Endo-LIF: Endoscopic lumbar interbody fusion
TLIF: Transforaminal lumbar interbody fusion
VAS: Visual Analogue Scale
ODI: Oswestry Disability Index
CT: Computed tomography
MRI: Magnetic resonance imaging

## References

[1] Mobbs RJ, Phan K, Malham G (2015). Lumbar interbody fusion: techniques, indications and comparison of interbody fusion options including PLIF, TLIF, MI-TLIF, OLIF/ATP, LLIF and ALIF. Spine Surg.

[2] Jin YM, Chen Q, Chen CY (2021). Clinical research and technique note of TLIF by Wiltse approach for the treatment of degenerative lumbar. Orthop Surg.

[3] Mu XP, Yu CQ, Wang CL (2021). Comparison of extreme lateral approach with posterior approach in the treatment of lumbar degenerative diseases: a meta-analysis of clinical and imaging findings. Surgeon.

[4] Liu HN, Li JQ, Sun YP (2022). A comparative study of a new retractor-assisted WILTSE TLIF, MIS-TLIF, and traditional PLIF for treatment of single-level lumbar degenerative diseases. Orthop Surg.

[5] Foley KT, Holly LT, Schwender JD (2003). Minimally invasive lumbar fusion. Spine.

[6] Song ZW, Zhu WH, Zheng JW (2022). Comparison of short-term efficacy of MIS-TLIF and Endo-LIF in the treatment of single-segment degenerative lumbar diseases. Front Surg.

[7] Lin EY, Kuo YK, Kang YN (2018). Effects of three common lumbar interbody fusion procedures for degenerative disc disease: a network meta-analysis of prospective studies. Int J Surg.

[8] Jitpakdee K, Liu Y, Heo DH (2023). Minimally invasive endoscopy in spine surgery: where are we now?. Eur Spine J.

[9] Wu AM, Hu ZC, Li XB (2018). Comparison of minimally invasive and open transforaminal lumbar interbody fusion in the treatment of single segmental lumbar spondylolisthesis: minimum two-year follow up. Ann Transl Med.

[10] Foley KT, Gupta SK, Justis JR (2002). Percutaneous pedicle screw fixation of the lumbar spine. J Neurosurg.

[11] Jin M, Zhang J, Shao H, Liu J, Huang Y (2020). Percutaneous transforaminal endoscopic lumbar interbody fusion for degenerative lumbar diseases: a consecutive case series with mean 2-year follow-up. Pain Physician.

[12] Kolcun JPG, Brusko GD, Basil GW, Epstein R, Wang MY (2019). Endoscopic transforaminal lumbar interbody fusion without general anesthesia: operative and clinical outcomes in 100 consecutive patients with a minimum 1-year follow-up. Neurosurg Focus.

[13] Jiang C, Yin S, Wei J (2021). Full-endoscopic posterior lumbar interbody fusion with epidural anesthesia: technical note and initial clinical experience with one-year follow-up. J Pain Res.

[14] Sairyo K, Morimoto M, Yamashita K (2021). Full-endoscopic trans-Kambin’s triangle lumbar interbody fusion: technique and review of literature. J Minim Invasive Spine Surg Tech.

[15] Ge DH, Stekas ND, Varlotta CG (2019). Comparative analysis of two transforaminal lumbar interbody fusion techniques: open TLIF versus Wiltse MIS TLIF. Spine.

[16] Khechen B, Haws BE, Patel DV (2019). Comparison of postoperative outcomes between primary MIS TLIF and MIS TLIF with revision decompression. Spine.

[17] Sayari AJ, You JS, Patel DV (2019). Device solutions for a challenging spine surgery: minimally invasive transforaminal lumbar interbody fusion (MIS TLIF). Expert Rev Med Devices.

[18] Zhang H, Zhou CL (2021). Percutaneous endoscopic transforaminal lumbar interbody fusion: technique note and comparison of early outcomes with minimally invasive transforaminal lumbar interbody fusion for lumbar spondylolisthesis. Int J Gen Med.

[19] Osman SG (2012). Endoscopic transforaminal decompression, interbody fusion, and percutaneous pedicle screw implantation of the lumbar spine: a case series report. Int J Spine Surg.

[20] Wang MY, Grossman J (2016). Endoscopic minimally invasive transforaminal interbody fusion without general anesthesia: initial clinical experience with 1-year follow-up. Neurosurg Focus.

[21] Lei X, Wu WJ, Yu L (2016). Comparison between minimally invasive transforaminal lumbar interbody fusion and conventional open transforaminal lumbar interbody fusion: an updated meta-analysis. Chin Med J.

[22] Jung JM, Son S, Yoo BR (2022). Full-endoscopic versus minimally invasive lumbar interbody fusion for lumbar degenerative diseases: a systematic review and meta-analysis. J Korean Neurosurg Soc.

[23] Ahn Y, Youn MS, Heo DH (2019). Endoscopic transforaminal lumbar interbody fusion: a comprehensive review. Expert Rev Med Devices.

[24] Son S, Ahn Y, Lee SG, Kim WK (2020). Learning curve of percutaneous endoscopic interlaminar lumbar discectomy versus open lumbar microdiscectomy at the L5–S1 level. PLoS ONE.

